# Conversational Agents and Avatars for Cardiometabolic Risk Factors and Lifestyle-Related Behaviors: Scoping Review

**DOI:** 10.2196/39649

**Published:** 2023-05-25

**Authors:** Lynnette Nathalie Lyzwinski, Mohamed Elgendi, Carlo Menon

**Affiliations:** 1 Menrva Research Group Schools of Mechatronic Systems Engineering and Engineering Science Simon Fraser University Metro Vancouver, BC Canada; 2 Faculty of Health Sciences Simon Fraser University Burnaby, BC Canada; 3 Biomedical and Mobile Health Technology Lab Department of Health Sciences and Technology ETH Zurich Zurich Switzerland

**Keywords:** chatbots, avatars, conversational coach, diet, physical activity, cardiovascular disease, hypertension, cardiometabolic, behavior change, hypertension diabetes, metabolic syndrome, mobile phone

## Abstract

**Background:**

In recent years, there has been a rise in the use of conversational agents for lifestyle medicine, in particular for weight-related behaviors and cardiometabolic risk factors. Little is known about the effectiveness and acceptability of and engagement with conversational and virtual agents as well as the applicability of these agents for metabolic syndrome risk factors such as an unhealthy dietary intake, physical inactivity, diabetes, and hypertension.

**Objective:**

This review aimed to get a greater understanding of the virtual agents that have been developed for cardiometabolic risk factors and to review their effectiveness.

**Methods:**

A systematic review of PubMed and MEDLINE was conducted to review conversational agents for cardiometabolic risk factors, including chatbots and embodied avatars.

**Results:**

A total of 50 studies were identified. Overall, chatbots and avatars appear to have the potential to improve weight-related behaviors such as dietary intake and physical activity. There were limited studies on hypertension and diabetes. Patients seemed interested in using chatbots and avatars for modifying cardiometabolic risk factors, and adherence was acceptable across the studies, except for studies of virtual agents for diabetes. However, there is a need for randomized controlled trials to confirm this finding. As there were only a few clinical trials, more research is needed to confirm whether conversational coaches may assist with cardiovascular disease and diabetes, and physical activity.

**Conclusions:**

Conversational coaches may regulate cardiometabolic risk factors; however, quality trials are needed to expand the evidence base. A future chatbot could be tailored to metabolic syndrome specifically, targeting all the areas covered in the literature, which would be novel.

## Introduction

### Background

Metabolic syndrome (MetS) is a highly prevalent condition that affects up to approximately 30% of adults aged >65 years worldwide [1]. It consists of multiple symptoms, namely abdominal obesity, glucose intolerance, hypertension, and high cholesterol as well as low high-density lipoprotein [2]. It is associated with a substantially increased risk of premature morbidity and mortality from diabetes and cardiovascular disease (CVD) [2]. Low levels of physical activity (PA) are strongly associated with MetS, including obesity and overweight [3], high blood pressure [4], and insulin intolerance [5]. Furthermore, low levels of activity are significantly associated with increased risk of complications of MetS, including diabetes and CVD [5,6]. In addition, research has found that losing weight by approximately 5% to 10% results in significantly reduced MetS-associated markers [1] in patients with existing disease, highlighting that MetS may be modifiable through lifestyle-related weight interventions. Dietary modifications, including reduced sodium, sugar, and fat intake, are also highly beneficial for reducing the risk of the syndrome and its complications [7].

In recent years, mobile health (mHealth) has increasingly been used to support behavior changes related to weight loss, including improving dietary intake and physical activity [8]. Research on the use of mHealth interventions has found support for a moderate effect size for assisting with weight loss [8]. This includes the use of SMS text messaging for behavior change and mHealth apps that target weight loss using a range of behavior change techniques (BCTs) [9], including self-monitoring, feedback, goal setting, education, tips, personal tailoring, reminders, encouragement, and social and professional support [8]. mHealth is a form of health care that enables timely accessibility, portability, and personalized medicine tailored to the needs of the user [10,11]. It includes smartphones, PDAs, MP3 players, iPads (Apple Inc), smart clothing, and smart watches [10,11].

Emerging research in the mHealth field has focused on developing conversational agents that can simulate human professional interactions for managing different health problems [12], including weight issues [13]. Furthermore, avatars have also been developed to display a conversational coach in addition to written conversational text, simulating real-life interactions with a professional, such as a live fitness coach [14,15]. Having a conversational coach complement or replace metabolic-related health advice from professionals may increase accessibility and enable more timely health monitoring and diagnosis of health conditions [15] such as MetS if physicians also gain access to patient data. Given that technology in the field is advancing, it is time to determine whether these conversational agents are effective for assisting with MetS-associated risk factors, including overweight, obesity, physical inactivity, and unhealthy dietary intake. There is also a need to better understand what types of weight-related and MetS-related studies have been undertaken using conversational agents and to identify challenges with the technology and future areas of research.

### Aims

This review aimed to better understand the evidence surrounding the use of conversational coaches for metabolic-related risk factors and biomarkers. Furthermore, this review aimed to determine whether conversational coaches are effective for improving weight-related behaviors and metabolic indicators and whether conversational agents are acceptable for consumers as agents of behavior change.

### Research Questions

- Research question (RQ) 1: How effective are conversational agents (chatbots and avatars) for weight-related behaviors, including diet and exercise?
- RQ 2: How effective are conversational agents for improving metabolic risk factors, including blood pressure, cholesterol, abdominal obesity, and glucose (diabetes management)?
- RQ 3: What are consumers’ perspectives on the use of chatbots?

## Methods

A systematic review of PubMed and MEDLINE was conducted in December 2021 for all relevant studies on conversational coaches for metabolic risk factors published over the last 10 years. Google Scholar was also searched for any additional papers along with manual hand searching.

### Inclusion and Exclusion Criteria

This review included studies on chatbots or avatar conservational agents that acted as coaches for improving metabolic health behaviors, including dietary intake (sodium and sugar intake), PA, and weight (including abdominal obesity). Studies that evaluated one or more physiological indicators of metabolic health or risk factors for MetS, such as diabetes, glucose intolerance, hypertension, cholesterol, and serum triglycerides, were also included. Studies must have been published in the English language to be included. Chatbots that were used for survey reasons but not primarily for targeting weight-related or metabolic risk factors were excluded. Studies whose primary focus was not on conversational coaches were excluded (including those that had an avatar element but did not primarily focus on evaluating it). Studies on wearables that did not include avatars or chatbots were excluded. Studies in pregnant women were excluded.

### Search

The keywords included word variations for “chatbot,” “virtual assistant,” “virtual coach,” or “avatar”; weight-related behaviors, including “diet,” “exercise,” or “weight”; and metabolic risk factors, including “hypertension,” “cholesterol,” or “diabetes.” The search strategy is shown in Textbox 1.

**PubMed search strategy example.**

**1.** **Cardiometabolic risk factors**

- Weight

“obesity”[MeSH Terms] OR “obese”[tiab] OR “obesity”[tiab] OR “overweight”[tiab] OR “overweight”[tiab] OR “BMI”[tiab] OR “Body mass index”[tiab] OR “Body mass index”[MeSH Terms] OR “physical activity”[Tiab] OR adiposity [tiab] OR weight gain[tiab] OR body weight[tiab] OR “abdominal visceral fat”[Tiab] OR “adipose tissue”[MeSH Terms] “weight loss”[Mesh] OR “weight loss”[tiab] or “metabolic syndrome”

- Diet and physical activity

diets[tiab] OR “diet”[mesh] OR diet[tiab] OR “energy intake”[tiab] OR nutrition[tiab] OR “diet, food, and nutrition”[MeSH Terms] OR diets[tiab] OR Caloric restriction[tiab]OR “physical activity”[tiab]

- Hypertension

hypertension[tiab] OR “Blood Pressure”[tiab] OR Prehypertension[tiab] OR BP[tiab] OR “Systolic blood pressure”[tiab] OR SBP[tiab] OR “Diastolic blood pressure”[tiab] OR DBP[tiab] OR cardiovascular[tiab] OR hypotensive[tiab] OR “Hypertension”[MeSH] OR “Blood Pressure”[MeSH] OR “Prehypertension”[MeSH]

- Cholesterol

“cholesterol”[MeSH Terms] OR cholesterol[tiab]

- Diabetes

“Diabetes Mellitus”[MeSH] or diabetes[tiab] or diabetic[tiab] or prediabetes[tiab] or pre-diabetes[tiab] OR “glucose”[MeSH Terms] OR “glucose”[tiab]

**AND**

**2. Technology**

chatbot*[tiab] OR chat bot[tiab] OR chat-bot[tiab] OR chatter bot[tiab] OR chat bots[tiab] OR chat-bots[tiab] OR chatter bots[tiab] OR chatterbot*[tiab] OR smart bot[tiab] OR smartbot[tiab] OR smart bots[tiab] OR smartbots[tiab] OR smart-bot*[tiab] OR virtual agent*[tiab] OR virtual character*[tiab] OR virtual coach*[tiab] OR virtual human[tiab] OR avatar*[tiab] OR embodied agent*[tiab] OR relational agent*[tiab] OR animated character*[tiab])

1 AND 2

### Screening and Data Extraction

Titles were screened for relevance to the RQs, followed by abstract and full-text retrieval of eligible studies that met the inclusion criteria. A second reviewer (LL) screened the abstracts and full texts against the inclusion and exclusion criteria to ensure agreement. Quantitative and qualitative data were extracted and summarized in a tabular format, including study characteristics, measures, outcomes, and intervention details.

## Results

### General Description

**Figure 1:**
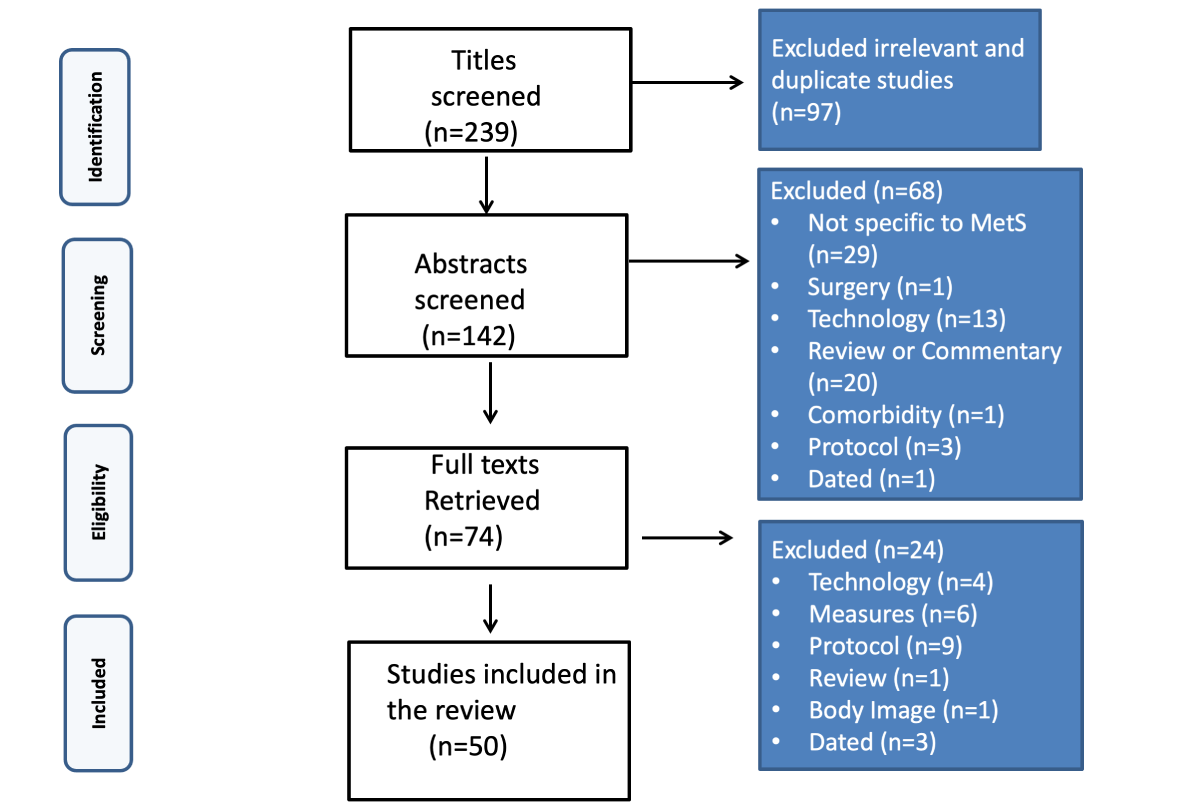
PRISMA (Preferred Reporting Items for Systematic Reviews and Meta-Analyses) flowchart of the search and screening process. MetS: metabolic syndrome.

LL and ME screened the final selected papers individually. A total of 52 full texts were selected [13,14,16-65]; however, after double peer screening, 1 protocol and 1 dated technology were removed. The final number included 50 papers [13,14,16-59,61-63,66]. Details of the search process and reasons for exclusion are illustrated in Figure 1 [67].

Most of the studies were feasibility and usability studies. A few studies were qualitative and explored consumer perspectives on conversational agents for weight-related behaviors [14,19]. The countries where the studies were conducted included Australia, the United States, Italy, Spain, and Taiwan [13,14,16-29]. Most of the studies explored virtual agents for diet and exercise, with only 2 (4%) exploring chatbots for hypertension management [17,19]. The majority were conducted among adults, but 3 (6%) were conducted among teenagers and preteens [14,26,29]. The study characteristics and results are summarized in Table 1.

**Table 1:** Study characteristics.

Study and year	Location, N, and design	Sex (%)	Age (years)	Health targets and measures	Technology and procedures	Outcomes
Echeazarra et al [17], 2021	Location: Spain N=112 Design: 2-year RCT^a^	Female: 42	Mean 52.1	BP^b^	Tensiobot (telegram app) Reminders to check BP Education on how to properly check BP using videos Warnings and graphic feedback on BP GPs^c^ can connect with the app to access patient data Advice offered 24/7	No significant differences in adherence between groups Bot group had higher levels of knowledge on good practice skills for BP (t=2.11; *df*=82.3; 95% CI 0.39-12.6; *P*<.05) Measurements (*P*<.05) Bot found to be acceptable/likable Adherence after intervention: 85%
Griffin et al [19], 2021	Location: United States N=15 Design: mixed methods questionnaires with semistructured interviews qualitative	Female: 53	Mean 59 (SD 11)	BP	Theoretical discussion around chatbots for hypertension medication management	Most patients were interested in and open to trying a chatbot for hypertension medication management and reminders Privacy concerns and usability with mobile phones
Larbi et al [20], 2021	Location: Switzerland N=30 Design: feasibility study	Female: 50	Range 18-69	PA^d^	MYA social media chatbot	Perceptions of usefulness and informativeness: 53% User friendly: 83% Failed to understand user input: 63.3% Potential confusion with using the technology 43.3%
Lin et al [27], 2021	Location: Taiwan N=96 Design: factorial experimental study with 4 arms	Female: 53	Mean 21.5; range 18-42	PA (core muscle exercise)	VR^e^ avatar	Increase in PA (vector movement) of 986.7 (SD 1.03) points in in normal realistic avatar relative to muscular avatar Higher self-efficacy for core muscle exercise in normal avatars vs muscular avatars in female participants (+0.66, SD 0.1 points) and higher levels than in male participants (+0.9, SD 0.2 points) *P*<.05
Dol et al [37], 2021	Location: The Netherlands N=71 Design: qualitative study	Female: 100	Mean 44.4 (SD 12.86); range 19-70	Emotional eating	Conversational coach for emotional eating	The design of the conversational coach should integrated dialectal behavioral coaching strategies, as preferred by participants with emotional eating behavior
Lin et al [27], 2021	Location: Taiwan N=104 Design: experimental design study	Female: 50	Mean 70.39 (SD 6.51); range 60-88	PA perceived exertion Self-efficacy	Assigned to either age-matched or young avatars for PA Theory: Proteus effect of avatar embodiment Watched videos in a digital gym where they exercised Wore a head-mounted display	Older male participants assigned to young avatars had higher perceived exertion than counterparts assigned to older ones (+1.56, SD 0.31 points; male participants only) Female participants assigned to young avatars had higher self-efficacy for future exercise than counterparts (+0.45 points) and male participants *P*<.05
Maher et al [13], 2021	Location: Australia N=31 Design: proof-of-concept study	Female: 67	Range 45-75	PA, Mediterranean diet, and weight	AI^f^ Paola chatbot teaches users about exercise and uses BCTs^g^, including goal setting, self-monitoring, and feedback	Mean increase in diet score: 5.7 (95% CI 4.2-7.3) Mean PA increase: 109.8 min (95% CI 1.9-217.9; *P*<.01) Mean weight loss: 1.3 kg (95% CI −2.5 to −0.7; *P*<.05) *P*<.01 No significant changes in blood pressure
Hickman et al [40], 2021	Location: United States N=109 Design: 2-arm RCT	Female: 59	Mean 52 (SD 11)	Hypertension, quality of the physician-patient interaction	Avatar intervention or video on hypertension management	Scores for the quality of the patient-provider interaction were better over time (F3=5.25; *P*<.01) in the within-subjects analysis along with a time by experimental condition interaction (F3=2.91; *P*<.05) Between-subject effects per treatment were insignificant No significant changes in blood pressure
Napolitano et al [49], 2021	Location: United States N=136 Design: feasibility study (12 weeks)	Female: 100	Mean 27.8 (SD 5.4)	Weight, diet, and PA; exercise self-efficacy	Conversational coach gave lessons on health behaviors	No significant results were found for differences in weight, PA, or consumption of fast food between the intervention arm and control groups High attrition 44% Goal achievement for nutrition <10%
Santini et al [55], 2021	Location: Austria, Italy, and Netherlands N=60 (2 waves) Design: qualitative study with focus groups and phone interviews	Female: 53.3% wave 1; 51.6% wave 2	Mean 61.9	Health behaviors, diet, and PA	Embodied coach for diet and PA	Desire for the avatar to motivate older adults to exercise Supportive tone and language that is not authoritarian or patronizing
Krishnakumar et al [44], 2021	Location: India N=102 Design: pre-post intervention (1 arm) 16 weeks	Female: 31.4	Mean 50.8	Diabetes (blood sugar), diet, PA, and weight (logged)	Wellthy CARE mobile app	The use of the Wellthy CARE digital therapeutic for patients with T2D^h^ showed a significant reduction in the mean levels of HbA_1c_^i^ −1.16% (95% CI −1.40 to −0.92; *P*<.01); FBG^j^ (−11 mg/dL), and PPBG^k^ (−22 mg/dL); *P*<.05 Weight decreased by 1.32 kg (95% CI −0.63 to −2.01 kg) after 16 weeks
Dhinagaran et al [36], 2021	Location: Singapore N=60 Design: one arm web-based feasibility stud	Female: 62	Mean 33.7	Diet, PA, sleep, and stress	Chatbot for diabetes prevention, diet, exercise delivered over Facebook Messenger (Meta Platforms Inc)	Engagement: 50% Retention: 93% Satisfaction: high at 92% 50% agreed that the chatbot was acceptable and usable No significant changes in health behaviors including PA Minimal improvement in diet: increase in fruit intake (3 portions) by 4% and vegetables once per day by 2%
To et al [61], 2021	Location: Australia N=116 Design: quasi-experimental study (6 weeks)	Female: 81.9	Mean 49.1 (SD 9.3)	PA	Fitbit plus a chatbot in the Messenger app	Usability score: 89.4% Desire to continue using: 35.4% Helped them: 53% Mean PA increase: 154.2 min/week (95% CI 2.28-5.63) OR for meeting PA guidelines: 6.37 (95% CI 3.31 to 12.27) Mean steps/day increase: 627 (95% CI 219 to 1035)
Mitchell et al [48], 2021	Location: United States N=158 Design: mixed methods survey with qualitative interviews study	Female: 100	Mean 56 (SD 11) intervention; 57 (SD 11) control	Diabetes	Avatar for diabetes self-management	Avatars provide support for diabetes self-management via 3 areas (self, social, and physical) that are linked with engagement
Strombotne et al [58], 2021	Location: United States N=590 Design: quasi-experimental study	Female: 11	Mean treatment=58.1; control=57.7	Diabetes and risk factors	Conversational coach and ketogenic diet	BP decrease (systolic): 1.4 mm Hg (95% CI −2.72 to 0.14) Diastolic BP levels decreased: −1.43 (95% CI −2.72 to −0.14) mm Hg HbA_1c_ decreased: −0.69 (95% CI −1.02 to 0.36) Diabetes medication fills: −0.38 (95% CI −0.49 to −0.26) BMI: −1.07 (95% CI −1.95 to −0.19) kg/m^2^
Alves Da Cruz [31], 2020	Location: Brazil N=27 Design: cluster randomized crossover trial	Female 48.1	Mean 63.4 (SD 12.7)	HR^l^, BP, and RR^m^	Avatars with exergames for PA in patients undergoing cardiovascular rehabilitation	Increase in HR (z=82.8; *P*<.01) and RR (z=12.9; *P*<.01) during and (5 min) after exergame Changes in systolic BP but not diastolic with differences within moments z=11.26 (*P*<.01) With no statistical significance between groups
Kowalska et al [43], 2020	Location: Poland N=249 Design: cross-sectional study	Female: 36.5	Mean 65.3 (SD 13.8)	CVD^n^	Telehealth voice technology with health professionals and voice conversational agent	High desirability for telehealth consultations with a cardiologist combined with a conversational agent Desirability for telemonitoring of vitals: 67.5% 70.7% wanted a consultation with a cardiologist remotely
Piao et al [51], 2020	Location: South Korea N=106 Design: Exploratory Randomized Controlled Trial 12 weeks	Female: 56 intervention; 57 control	Range 20-59	Health behaviors (diet and exercise); SRHI^o^	Lifestyle coaching chatbot Informed by habit formation Cues and goals	Significant improvement in health behavior The intervention group had higher scores on the SRHI of 7.12 (SD 5.57) with *P*<.05 at 4 weeks; no significant differences between groups at 12 weeks, PA remained higher after 12 weeks (*P*<.05)
Naylor et al [50], 2020	Location: United States N=20 Design: pilot study	N/A^p^	Mean 8.4 (SD 1.3)	VO_2_ (mL × kg^–1^ × min^–1^) using indirect calorimetry questionnaire on liking and motivation	Children played tennis with their friend and an avatar	Increased VO2 during game play in both cooperative (3.8 + 1.8 mL × kg^-1^ × min^-1^) and competitive play (4.4 +1.8 mL × kg^-1^ × min^-1^) compared with resting condition (*P*<.01) Children liked exercising more in cooperative games than in competitive games (*P*<.01) No differences between game styles in motivation for PA (*P*>.05)
Hahn et al [39], 2020	Location: United States N=42 (child and parent dyads [n=40 completed baseline and follow-up measures]) Design: pilot intervention	Female (children): 55.2	Treatment: mean 8.06 (SD 1.10); control: mean 7.5 (SD 1.38)	PA using Fitbit and self-report on motivation for PA	Children wore Fitbit with a personalized dog avatar for socializing and support (digital fitness kiosk); theory informed (social cognitive theory)	Completion rate: 81.63% Mean number of PA goals reached: 3.28 Mean time playing with pets: 20.35 min Mean number of active min: 66 min (no statistical significance was found)
Navarro et al [24], 2020	Location: United States N=305 Design: 3-arm RCT	Female: N/A	Mean 20.0 (SD 2.2); range 18-37	Cardiac frequency, step counts, accelerometer, and HR monitor	Randomly assigned to avatars embodying them (same face) or different from them (strangers) Avatars wore normal clothes or gym clothes	Higher cardiac output (frequency) from 6 to 12 min in users of avatars that had a similar appearance (face) Higher output in users with avatars that additionally wore sports clothing at 6-7 and 10-minute periods Support for the Proteus effect hypothesis No changes in step count
Davis et al [16], 2020	Location: Australia N=28 Design: pilot single-arm study	Female: 68	Mean 56.2 (SD 8); range 45-75	Diet: Mediterranean diet adherence tool. Weekly log for diet and step count; activity tracked using a wrist worn tracker (Garmin) that syncs with Paola. Minutes of moderate to vigorous PA assessed with Active Australia Survey	Conversational assistant Paola for diet and PA consisted of educational modules, weekly check-ins, and 24/7 availability for PA and diet questions 12-week pilot	Assisted with increasing PA (step goal achieved 59% of the time) Adherence to diet: 91%
Navarro et al [23], 2020	Location: Spain N=42 Design: 3 arms—2 avatars vs control	Female: 100	Mean 31.9 (SD 11.7); range 19-61	PA, IPAQ^q^, self-efficacy to regulate exercise, and PA enjoyment scale (PACES^r^)	Avatar: ideal (perfect body) or normal (matching the participant) and web-based intervention without the avatar	Increased PA in all groups (F1,39=15.8; *P*<.01; web-based intervention effects) No effects of time by avatar assignment, ie, interaction
Balsa et al [32], 2020	Location: Portugal N=20 Design: usability study with qualitative interviews	Female: experts 88.9%; end users 27.3%	Mean 62.62; mean end users 70.9; mean experts 54.3	Usability of the app for diabetes medication adherence and improving lifestyle behaviors, diet, and PA	The conversational coach resembles a human Integrated BCTs: goal setting, self-monitoring, feedback, and social support/counseling	Usability score: 73.75 (SD 13.31) (indicates high usability of the coach)
Chin et al [35], 2020	Location: United States N=15 Design: feasibility study	Female: 60%	Mean 67 (SD 5.84)	PA	Health coach for PA As part of a PA program using a Google Home device (Google LLC)	Usability was high 80% of the participants did not experience challenges when interacting with the conversational coach
Fadhil et al [18], 2019	Location: Italy N=19 Design: validation study (4 weeks)	Female: 42	Mean 28.5; range 19-53	Diet and PA questionnaires via chatbot and motivation (HAPA^s^)	CoachAI text based conversational agent Tailored coaching for habits	Participants were satisfied with the agent High trust to share personal information to the coach
Ahn et al [30], 2019	Location: United States N=67 Design: field study (3 days)	Female: 61.19	Mean 11.24 (SD 0.85); range 9-13	PA and basic psychological needs	Use of a digital dog, with and without a points-based reward system	Higher levels of PA in the rewards points group briefly versus control (F1,58=5.32; *P*<.05) No significant effects on PA over time
Stephens et al [26], 2019	Location: United States N=23 Design: feasibility study	Female: 57	Mean 15.2; range 9.7-18.5	Weight management; pre-diabetes	Tess text-based chatbot counsellor for healthy behavior change usability assessed with progress toward goals and engagement	Usefulness rate: 96% Progress toward goals frequency: 81%
Srivastana et al [57], 2019	Location: United States N=10 Design: usability study	Female: 70	Range 44-67	Prediabetes	The web-based module used to support diabetes prevention education and a mobile app that is an electronic diary and a coach	Success of modules 60% as they meet weight loss of 5% Compliance with dietary recommendations: 59%-87% Compliance with PA: 52%-93%
Thompson et al [59], 2019	Location: United States N=27 Design: pilot feasibility study	Female: 73 (teens)	Range 10-15	Diabetes	Conversational agent with human features Conversations around diabetes education	Attrition: low (<10%) High satisfaction: >80% Technical issues<10% Teens and families had a positive experience
Thompson et al [29], 2018	Location: United States N=48 Design: laboratory-based study	Female: 50	Range 12-14	PA	PA exergame with an avatar coach	Completion: 87.5%; teens enjoyed the game (mean enjoyment score 68%) Vigorous PA during 74.9% of the game
Duncan-Carnesciali et al [38], 2018	Location: United States N=198 Design: cross-sectional, survey-based design using quantitative and qualitative paradigms	Female: 97.5	Range 26-76	Diabetes	Avatar for diabetes management	Ethnicity including Arab or Middle Eastern, Asian, and White or European descents as well as age were significantly associated with an excellent rating of the video with *P*<.05
Klaassen et al [42], 2018	Location: N/A N=21 Design: usability study	Female: 52	Mean 13.9	Diabetes	Conversational coach game with feedback Integrates BCTs including information on consequences	Usability index of 44.18 (SD 21.18; low)
Sinoo et al [56], 2018	Location: Netherlands N=21 Design: experimental study	Female: 37	Mean 9.2 (SD 1.1)	Diabetes self-management	Avatar for gameplay and diabetes self-management vs robot	Preference for the robot (mean friendship score 4.0, SD 0.6) over the avatar (mean friendship score 2.9, SD 0.7) as a companion Usability moderate: 58.7 (SD 24.5) Similarity of avatar to robot led to greater friendship (*P*<.01)
Tongpeth et al [62], 2018	Location: Australia N=22 (development of the application) N=10 (feasibility testing) Design: pilot feasibility	Female: 10	Mean 52.2 (SD 10.4)	Cardiovascular: acute coronary syndrome management	An interactive, avatar-based education application for improving patients’ knowledge of, and response to, acute coronary syndrome symptoms	Symptom recognition increased: 24% Satisfaction: 87.3% Knowledge increase: 15.7%
Friedrichs et al [63], 2014	Location: Netherlands N=958 Design: 3-arm RCT	Female: 60.4	Mean 42.9 (SD 14.5)	PA; Dutch Short questionnaire	Avatar with a web intervention or a digital web-based text condition versus control	Significant increases in PA in the intervention arms versus control with B=0.39 in the avatar arm and B=0.44 in the text arms (*P*<.05) No differences between the text arm or the avatar arm for PA
Stein et al [25], 2017	Location: United States N=70 Design: longitudinal observational study	Female: 74.5	Mean 47 (SD 1.8); range 18-76	Weight and dietary intake	Lark Weight Loss Health Coach (participants were a part of a diabetes prevention weight loss program) Advice on dietary intake and PA BCTs used include motivation, encouragement, reminders, and education	31% increase in healthy eating Mean weight change: −2.4 kg (SE 0.82; 95% CI −4.03 to −0.77)
Thompson [66], 2016	Location: United States N=48 (round 1) N=43 (round 2) Design: mixed methods survey with qualitative interviews	Female: 50	Range 12-14	Preferences for a PA intervention	Exergame with a self-representation avatar	Desired gameplay with the avatar that could be controlled by eliciting the desired action: 62.5% male and 58.3% female Personalized avatar: 41.7% Most common avatar features to be customized: Body: 95.8% Clothing: 93.8% Hair color: 87.5%
Behm-Morawitz et al [33], 2016	Location: United States N=90, female=100% (the 2 male participants were excluded) Design: qualitative research and RCT	Female: 100	Range 18-61	Weight and PA self-efficacy	Avatar (embodied) and video game to promote PA	Findings support the use of the avatar for weight management *t*_18_=2.15 (*P*<.05) with the intervention losing 1.75 lbs versus 0.91 lbs in the control No effects on dietary self-efficacy Strong correlation with avatar sense of self-presence and confidence in meeting health goals (r=0.95; *P*<.01) Themes: avatar benefits include motivation and assisting with self-efficacy for PA Barrier: games are not for everyone
Kuo et al [45], 2016	Location: Taiwan N=76 Design: 2-arm intervention in laboratory	Female: 63.15	Mean 21.2	Eating behavior observed in laboratory	Avatar that embodied the participants or was a weight-reduced (thinner) version of them	Avatars that embodied a thinner version of the participants shaped eating behaviors more compared with identical self-avatars; including selecting less ice cream (Cohen d=0.35; F1,73=7.8; *P*<.01) and opted for sugar free drinks (Cohen d=0.29; F1,73=6.0; *P*<.01)
Ruiz et al [54], 2016	Location: United States N=41 Design: laboratory study	Female: 0	Mean 64 (SD 7)	Cardiovascular behavioral risk factors (diet and exercise)	Avatar vs a voice (nonanimated) for behavior change linked with CVD	Avatar increased intentions (+1.56 points) to improve lifestyle behaviors relative to controls (Cohen d=0.77 *P*<.01; *t*_36_=2.48) Differences in confidence to change risk of heart disease was nonsignificant
LeRouge et al [14], 2015	Location: United States N=41 Design: user-centered design, 3 phases with focus group and interviews	N/A	Teenagers: 12-17	Perceptions of the avatar for diet and exercise	Interactive avatar coach	Desire for a fun human-like interaction Desire for a lifestyle coach and personal embodiment avatar and an authoritarian one Desire for customization of the avatar Advice on activity on the go and meals when eating at home Goal setting Technical issues could be a barrier including the internet
Thomas et al [28], 2015	Location: United States N=37 Design: feasibility and usability study with pre-post test	Female: 100	Mean 55.0 (SD 8.2)	Weight-related eating behaviors	Conversational coach for weight (focuses on dietary intake and managing eating behaviors)	The coach assisted with perceptions of increased self-control over eating (confidence to control eating: +1 point (SD 0.2; *P*<.01) and skills for controlling eating +0.7 points (SD 0.1; *P*<.01)
Ruiz et al [53], 2014	Location: United States N=150 Design: RCT	Male: 100	Mean 62 (SD 7.9)	Diabetes (knowledge)	Computer program with an avatar to increase diabetes knowledge and medication (adherence)	There were no significant differences between the intervention group and control group in terms of knowledge, with *P*=.95 Satisfaction levels were higher in the digital intervention group (F4=3.11; *P*<.01)
Li et al [46], 2014	Location: Singapore N=140 Design: factorial design experiment	Female: 41	Range 9-12	PA attitudes, motivation, and game performance	Assigned to varying avatars (normal and overweight)	Healthy weight avatars linked with greater scores in motivation for Nintendo exercise (F1,134=5.49; *P*<.05 [boys]) attitude, and performance (F1,134=2.27; *P*<.05 [girls])
Napolitano et al [22], 2013	Location: United States N=128 (phase I) N=8 (phase 2) Design: mixed methods (pilot usability testing) study with interviews	Female: 100	Mean 34.1 (SD 13.0); range 18-60 (phase 1)	Weight, PA [14], and weight self-efficacy; satisfaction; preferences survey and interviews	Avatar for diet and exercise Informed by social cognitive theory Behavioral modeling Targeted self-efficacy 4 weeks	The avatar helpful: 87.5% Mean weight loss after 4 weeks: 1.6 (SD 1.7) kg All women found that it helped with their diet and exercise Most were interested in the avatar
Bickmore et al [34], 2013	Location: United States N=122 Design: 4-arm RCT (2 months)	Female: 61	Mean 33.0 (SD 12.6); range 21-69	Diet (NIH^t^/NCI^u^ fruit and vegetable scan) and PA (IPAQ)	Animated counselor for diet and PA (separate and combined)	No significant differences between groups in PA after adjustment Fruit and vegetable servings significantly increased in the diet arm (F3,103=4.52; *P*<.01) No significant differences in weight or PA between groups Likability: Karen was perceived as nice by 35% of the participants 50% of the participants found Karen helpful
Johnoson-Glenberg et al [41], 2013	Location: United States N=19 Design: pilot feasibility study (pre-post study)	N/A	Grades 4-12 (ages 9-18)	Diet (nutrition and food choice test and knowledge)	Diet and exercise game (exergame) with an alien interactive coach	Differences in dietary knowledge of nutrition pre and post intervention (t=4.13; *P*<.01) and knowledge of the My Plate content in the study (t=3.29; *P*<.01)
Ruiz et al [52], 2012	Location: United States N=30 Design: comparative pilot with three arms (with randomization) intervention	N/A	N/A	PA	3D avatar-based VR intervention	Participants completing a 3D VR intervention mediated by avatars resembling the participants showed significant improvement in PA (*P*<.05) No significant effects of the intervention on obese or overweight participants
Mestre et al [47], 2011	Location: France N=6 Design: laboratory experimental study	N/A	Range 19-25	PA enjoyment	Digital coach paced participants in a VR bicycling setting	The VR coach and VR cycling were associated with higher levels of PA enjoyment (F2,10=13.24; *P*<.001) in the feedback group

^a^RCT: randomized controlled trial.

^b^BP: blood pressure.

^c^GP: general practitioner.

^d^PA: physical activity.

^e^VR: virtual reality.

^f^AI: artificial intelligence.

^g^BCT: behavior change technique.

^h^T2D: type 2 diabetes.

^i^HbA_1c_: hemoglobin A_1c_.

^j^FBG: fasting blood glucose.

^k^PPBG: postprandial blood glucose.

^l^HR: heart rate.

^m^RR: respiratory rate.

^n^CVD: cardiovascular disease.

^o^SRHI: Self-Report Habit Index.

^p^N/A: not applicable.

^q^IPAQ: International Physical Activity Questionnaire.

^r^PACES: physical activity enjoyment scale.

^s^HAPA: Health Action Process Approach.

^t^NIH: National Institutes of Health.

^u^NCI: National Cancer Institute.

### Weight

A few studies evaluated the effects of conversational assistants for weight loss [13,22-24,44]. The study by Maher et al [13] in Australia found that the conversational assistant (chatbot) Paola assisted with a weight loss of 1.3 kg at 12 weeks follow-up (95% CI –0.1 to –2.5). In addition, there was a mean waist circumference reduction of 2.5 cm at follow-up compared with baseline (95% CI –3.5 to –0.7). The chatbot used a range of BCTs, including goal setting, self-monitoring, education, social support, and feedback to users on PA and the Mediterranean diet [13]. A study in the United States found that the Lark Weight Loss Coach, an artificial intelligence–powered bot, assisted participants with a weight loss of 2.38% (95% CI –3.75 to 1.0) with a mean use of 15 weeks [25]. The conversational agent was informed by cognitive behavioral therapy and used a range of BCTs, including education, encouragement, and reminders surrounding dietary and PA targets [25]. The determinants of weight loss included the duration of using the artificial intelligence program and engaging with it, logging meals, and the number of counseling sessions completed [25]. A large study in the United States examining the use of an avatar coach that targeted self-efficacy and modelled vicarious experiences for diet and PA (4 weeks) found that women lost an average of 1.6 (SD 1.7) kg at follow-up [22]. A study in India found that an avatar coaching app with calls from health professionals assisted with a weight loss of 1.39 kg (95% CI –0.63 to –2.01; *P*<.01) at 16 weeks [44]. A randomized controlled trial (RCT) with a qualitative component found that avatars increase motivation and PA self-efficacy linked with weight loss [33]. However, some studies did not report any significant weight loss [34,49].

### Diet

A few studies evaluated the effects of conversational coaches (chatbots and avatars) on dietary intake and found that overall, the coaches assisted with ameliorating dietary habits and goals [13,16,25,28,34,45,49]. A study in the United States found that healthy dietary intake improved in 30% of participants who were using a conversational weight loss coach [25]. Another study found that eating behaviors improved in users of a conversational eating coach, which included increases in the mean scores for the perceptions of skills to eat healthily and self-control over their eating habits (0.7 increase in points) as well as confidence to control food consumption in social situations (1.0 increase in points; *P*<.01) [28]. The Paola chatbot study found a mean increase in the Mediterranean diet score [68] of 5.8 points at 12 weeks follow-up [13]. Similarly, a study of Karen, an animated counselor, found significant increases (*F*_3,103_=4.5; *P*<.01) in fruit and vegetable intake in the diet intervention arm relative to the control group [34]. A further study found that eating behaviors were shaped by the appearance of the avatar, with healthier eating behavioral patterns in participants who had thinner avatars including reduced portions of ice cream and opting for healthier sugar-free drink alternatives [45].

### Physical Activity

A few conversational assistant PA coaches, including chatbots and avatars, were evaluated, and overall, they assisted with increasing PA [13,16,21,23,24,27,55,63]. Most of them involved exergames with the avatar. However, one of the studies did not find any improvements in PA among the 2 avatars, attributing improvements only to the web-based part of the intervention [23], and another study did not find a difference between the web-based intervention and the chatbot (only when considering a standard control) [63]. A preliminary usability study in Australia found that step count goals increased 59% of the time in users of the chatbot that targeted PA and that participants had a preference for personalization and greater knowledge-based content [16]. Another pilot study of Paola, the chatbot in Australia, found that it assisted with increasing mean step count by 109 minutes per week at 12 weeks follow-up (95% CI 1.9-217.7) [13]. A study involving an exergame that used a PA avatar coach in teens found that 75% of the time, participants engaged in 15.88 (SD 5.8) minutes of vigorous PA throughout the game [29]. Participants also wanted the avatar to have a supportive and nonpatronizing or nondisparaging tone in interactions regarding PA and found that it could motivate older adults when adequately personalized [55]. Similarly, a study in children also found that they desired the option to personalize the avatar, including controlling and customizing its physical appearance during game play when exercising [66].

### Proteus Effect

The Proteus effect is a phenomenon wherein individuals embody and emulate the behaviors of their virtual characters such as avatars [69,70]. A few studies demonstrated support for the Proteus effect when it came to PA behaviors, although the type of avatar varied. A study in Taiwan found that younger looking avatars were associated with higher levels of PA than older looking avatars but only in women. Male participants had higher levels of PA than female participants who used an older looking avatar, highlighting differences between sexes [27]. A further study found a higher cardiac output resulting from increased intensity of PA in adult users of an avatar that resembled them and wore gym clothes when compared with avatars that appeared unfamiliar like strangers in regular clothing, which reduced heart rate [24]. Similarly, a study in Taiwan found increases in physical activity assessed in movements (986.7 points higher) in users of a “normal avatar”, more closely resembling them than the most muscular avatar [21]. They also found that self-efficacy was higher (0.66 points) for core muscle exercises in female participants assigned to normal avatars relative to their muscular counterparts and male participants assigned to the same standard avatar (0.9 points higher), with *P*<.05 [21]. Similarly, dietary behavior was also shaped by thinner embodied avatars in another study [45].

### Diabetes

Most diabetes studies were feasibility studies. The results of diabetes conversational coaches were mixed. A few studies did not have positive findings concerning the applications with avatars for diabetes [42,53]. However, one study reported a usability score of 73, which is relatively high. Notably, the study integrated a range of BCTs, including goal setting, feedback, self-monitoring, social support, and counseling [32]. Low usability scores were reported in a few studies, including one that reported an overall score of 44.58 (SD 21.18) [42]. Similarly, an RCT of a diabetes coaching avatar did not find that knowledge increased relative to controls, but intervention participants in the computer-based programmed dynamic avatar had higher satisfaction levels (*F*_4_=3.11; *P*=.01) [53]. Another study in the United States in participants with prediabetes found that 60% of patients had successfully completed the modules and met weight targets during 6 months of use (60% success rate) [57]. Engagement was also moderately high (50%) in a study in Singapore involving a chatbot, although usability was high along with retention (93%) [36]. In a study in teenagers, attrition was also low, and 80% of the participants were satisfied with the conversational diabetes coach [59]. A study that evaluated a coaching application for diabetes found an improvement of –11 mg/dL in fasting blood glucose levels [44]. However, the intervention also involved phone calls from health professionals [44]. Similarly, an avatar application with a ketogenic diet program assisted with a reduction in hemoglobin A_1c_ levels of 0.69% (SE 0.168%) [58]. Qualitative research found that avatars created an environment of social presence that facilitated social support and coherence for patients with diabetes [48]. In another study of avatars combined with robots, children preferred robots over avatars, but their friendship increased if the two had a greater similarity, which impacted usability [56].

### CVD and Associated Risk Factors

A few studies evaluated the use of conversational coaches for CVD. One of them was a pilot study of the Tensiobot chatbot [17], a coaching application that teaches users how to properly check their blood pressure using recommended practice guidelines and provides users with graphic feedback and reminders. The study found that the chatbot group did not differ from the control group in terms of adhering to blood pressure measurement recommendations. However, there were significantly higher levels of knowledge (+6.53 points) with regard to checking blood pressure in the chatbot group than in the control group (*P*<.05) [17]. Blood pressure (diastolic) was significantly reduced, that is, by 1.43 mm Hg (SE 0.65; 95% CI –2.72 to –0.14; *P*<.01), in users of an avatar application that also involved a ketogenic diet [58]. In addition, a mixed methods study with a qualitative component found that users in general were interested in trying a hypertension chatbot for medication management as well as for health communication and self-care [19]. In addition to these studies, a general diet and PA chatbot study evaluated changes in blood pressure, but these changes were nonsignificant [13]. A study in Poland found high desirability for a CVD voice technology coach, in addition to accessing phone-based telemedical services by health professionals [43]. A further study in Brazil evaluated avatars for cardiovascular rehabilitation and found that an avatar with an exergame influenced heart rate, systolic blood pressure, and respiratory rate during the intervention and up to 5 minutes after its completion [31]. Furthermore, a study found that the avatar intervention increased the intent to improve lifestyle behavioral risk factors in patients relative to controls (*P*=.01), although confidence did not change [54]. Finally, a study evaluated a cardiovascular educational avatar application and found that it increased symptom recognition by 24% and knowledge of CVD by 15%, with a high satisfaction rate of 87% among patients [62].

### User Perceptions

Several studies found that users were interested in using conversational coaches for lifestyle behaviors [14,19,22]. Overall, participants enjoyed using the chatbots and avatars or found them helpful for diet, exercise, and hypertension management [17,22,25,26,29]. User-friendliness was reported by 83% of the participants in a study that evaluated a PA social media chatbot [20]. Similarly, 87.5% of women in a weight loss avatar intervention found it helpful [22]. With the exception of studies on diabetes conversational coaches, adherence or completion of tasks was high across studies on lifestyle (diet and PA) conversational coaches, ranging from 85% to 90% [13,16,17,29]. The qualitative study themes were related to the desirability for a conversational coach for hypertension and weight-related behaviors, especially for one that simulates human interaction closely, provides advice and goals for meals when cooking, and provides educational support [14] including for hypertension management [14,19].

### Technological Challenges

A few tech challenges were brought up across the studies. Although users found that the conversational coach answered basic questions correctly, failure to understand and respond to more complex or spontaneous questions was reported in the studies. The percentage of failure for spontaneous or complex questions was 79% in one study [16], and participants in another study gave a high ranking for the chatbot’s failure to recognize their input [20]. Paola chatbot correctly answered spontaneous questions on diet in 4 out of 20 attempts, with a success of 20%, while the percentage of correctly answered simple and predetermined questions and responses was 96% and 97% [16].

## Discussion

### Principal Findings

This review aimed to better understand the effectiveness of virtual coaches for managing metabolic health and weight-related risk factors. It appears that virtual coaches hold potential for assisting patients with improving their dietary intake and PA behaviors, leading to subsequent weight loss. However, more studies that are larger and sufficiently powered RCTs are needed to establish a stronger evidence base. RCTs are the gold standard of evidence but are often costly and time-consuming [71,72]. Most of the studies were limited, as they were pilot studies. Ideally, it would be of interest to research long-term weight changes and cardiometabolic risk factor modifications over longer periods.

It appears that PA interventions may benefit from using avatars that embody the participant. The Proteus effect is based on the hypothesis that users adjust their behavior by modeling the virtual character with which they interact [73]. Thus, it seems that incorporating an avatar may enhance mHealth chatbot interventions, as it adds the element of user interaction and promotes the modeling of behavior through embodiment [73]. However, 1 (2%) study did not find that the avatars enhanced the effects of the web-based intervention [23].

We also found that consumers seemed to be interested in and enthusiastic about trying virtual coaches for managing their weight-related behaviors and blood pressure. Adherence to the intervention was also high throughout the studies, which indicates that this technology is acceptable and usable for patients. However, there is a need to undertake qualitative research on developing a MetS coach to further understand consumer perspectives. The main barrier to consider when developing future virtual agents is that the virtual agents did not always answer correctly to spontaneous responses. As consumers want personalized and tailored mHealth for weight-related behaviors [74], future applications should ensure that the virtual agents are sufficiently advanced to be able to interact with users in a natural and personalized manner.

It appears that diabetes virtual coaches should be improved to maximize engagement and adherence, as not all studies found that they were helpful. Although outside the scope of this review, we note that some studies used BCTs, which could suggest that future applications may benefit from integrating BCTs [13,20,22,25,32,42,51]. In addition, we identified some studies on blood pressure and CVD management, which demonstrated preliminary improvement in patients with hypertension as well as knowledge of CVD. However, we did not identify any virtual coaches for managing MetS. Therefore, there is a need to develop virtual coaches specifically tailored to this syndrome and its associated risk factors. Such virtual coaches could be integrated into a combined synchronized application that involves diabetes and CVD education and monitoring.

MetS is linked with high blood pressure, which is one of the main hallmarks of the disease. The theoretical mechanisms underpinning the development of hypertension in patients with MetS have included a combination of endothelial dysfunction, systemic inflammation, adiposity, and oxidative stress [75]. Dysfunction in the renin-angiotensin system has also been theorized to be a determinant [75]. Obesity itself has also been identified as a risk factor for high blood pressure in MetS [76]. Blood pressure is modifiable to some extent through lifestyle changes previously described, including dietary sodium restriction, PA, stress reduction [77], and medication [78]. Future virtual coaches may target hypertension as part of a MetS intervention, and this review found that patients are willing to try chatbots for managing their blood pressure.

MetS is also associated with high glucose levels of at least 100 mg/dL when patients are fasting [78], which indicates that they are in the prediabetes stage, as diabetes begins at fasting glucose levels of 126 mg/dL [79]. In a recent longitudinal study, patients who reduced their fasting blood glucose levels decreased their overall risk of diabetes by 54% when compared with their counterparts who did not improve their blood sugar levels (95% CIs exclude 1) [80]. A recent study found that individuals who consumed high amounts of sugar were 32% more likely to have MetS than their counterparts [81]. Thus, a future MetS virtual coach could target blood glucose monitoring and offer personalized advice on optimum sugar intake.

In addition to targeting dietary intake, PA is integral to managing this syndrome. A meta-analysis found that the risk of cardiovascular events was reduced by 30% in physically active individuals compared with those who were inactive [6]. A longitudinal study in middle-aged women found that increasing step counts significantly reduced, by 30%, the risk of MetS in this population and that they had clinically improved levels of the protective cholesterol high-density lipoprotein, whereas their serum triglycerides had significantly decreased [82]. A review found that walking on a daily basis reduced the risk of type 2 diabetes by nearly half [5]. Furthermore, recent research suggests that sedentary behavior, including sitting time, is an independent and significant risk factor for MetS syndrome [83]. Thus, PA chatbots and avatars, which were found to increase PA time, steps, and self-efficacy in this review, could be integrated into a comprehensive future MetS interventions.

Given that chatbots and avatars hold potential for increasing PA and reducing sedentary behavior, as well as improving dietary intake, studies are needed to evaluate their effectiveness for managing the symptoms and risk factors associated with MetS specifically.

In addition, stress is often an underlying determinant of maladaptive weight-related behaviors, including binge eating, emotional eating, and an unhealthy dietary intake as well as weight gain [84-88]. Future avatar and chatbot interventions for cardiometabolic factors could also consider integrating psychological supportive interventions such as mindfulness-based stress reduction, which assists with weight and stress [89-93], as an element.

### Conclusions

In summary, we found that virtual coaches hold promise for regulating diet, PA, weight, and possibly hypertension. However, studies on virtual coaches are few in number; therefore, more research, including RCTs, is needed to confirm the effectiveness of virtual coaches. Overall, most participants in the reviewed studies were interested in using virtual coaches, including chatbots and avatars, for regulating their weight-related behaviors, and study adherence was good. Future interventions could be ameliorated to reduce technical challenges associated with these conversational agents and ensure that they respond correctly to complex and spontaneous questions. Furthermore, future research could involve developing a comprehensive conversational agent for MetS, such as a health coach that simultaneously targets diet (sodium, sugar, and fat intake), exercise, weight (including abdominal obesity), blood pressure, and diabetes, and evaluating it. This would include a health coach that simultaneously targets diet (sodium, sugar, and fat intake), exercise, weight (including abdominal obesity), blood pressure, and diabetes.

## Abbreviations

BCT: behavior change technique
CVD: cardiovascular disease
MetS: metabolic syndrome
mHealth: mobile health
PA: physical activity
RCT: randomized controlled trial
RQ: research question

## References

[1] Han TS, Lean ME (2016). A clinical perspective of obesity, metabolic syndrome and cardiovascular disease. JRSM Cardiovasc Dis.

[2] (2021). About metabolic syndrome. American Heart Association.

[3] Wiklund P (2016). The role of physical activity and exercise in obesity and weight management: time for critical appraisal. J Sport Health Sci.

[4] Hegde SM, Solomon SD (2015). Influence of physical activity on hypertension and cardiac structure and function. Curr Hypertens Rep.

[5] Hamasaki H (2016). Daily physical activity and type 2 diabetes: a review. World J Diabetes.

[6] Li J, Siegrist J (2012). Physical activity and risk of cardiovascular disease--a meta-analysis of prospective cohort studies. Int J Environ Res Public Health.

[7] Hoyas I, Leon-Sanz M (2019). Nutritional challenges in metabolic syndrome. J Clin Med.

[8] Lyzwinski LN (2014). A systematic review and meta-analysis of mobile devices and weight loss with an intervention content analysis. J Pers Med.

[9] Michie S, Richardson M, Johnston M, Abraham C, Francis J, Hardeman W, Eccles MP, Cane J, Wood CE (2013). The behavior change technique taxonomy (v1) of 93 hierarchically clustered techniques: building an international consensus for the reporting of behavior change interventions. Ann Behav Med.

[10] (2011). mHealth: new horizons for health through mobile technologie. World Health Organization Global Observatory for eHealth.

[11] Lefebvre C (2009). Integrating cell phones and mobile technologies into public health practice: a social marketing perspective. Health Promot Pract.

[12] Griffin AC, Xing Z, Khairat S, Wang Y, Bailey S, Arguello J, Chung AE (2020). Conversational agents for chronic disease self-management: a systematic review. AMIA Annu Symp Proc.

[13] Maher CA, Davis CR, Curtis RG, Short CE, Murphy KJ (2020). A physical activity and diet program delivered by artificially intelligent virtual health coach: proof-of-concept study. JMIR Mhealth Uhealth.

[14] LeRouge C, Dickhut K, Lisetti C, Sangameswaran S, Malasanos T (2016). Engaging adolescents in a computer-based weight management program: avatars and virtual coaches could help. J Am Med Inform Assoc.

[15] (2021). Virtual trainer. Charamel.

[16] Davis CR, Murphy KJ, Curtis RG, Maher CA (2020). A process evaluation examining the performance, adherence, and acceptability of a physical activity and diet artificial intelligence virtual health assistant. Int J Environ Res Public Health.

[17] Echeazarra L, Pereira J, Saracho R (2021). TensioBot: a chatbot assistant for self-managed in-house blood pressure checking. J Med Syst.

[18] Fadhil A, Wang Y, Reiterer H (2019). Assistive conversational agent for health coaching: a validation study. Methods Inf Med.

[19] Griffin AC, Xing Z, Mikles SP, Bailey S, Khairat S, Arguello J, Wang Y, Chung AE (2021). Information needs and perceptions of chatbots for hypertension medication self-management: a mixed methods study. JAMIA Open.

[20] Larbi D, Gabarron E, Denecke K (2021). Social media chatbot for increasing physical activity: usability study. Stud Health Technol Inform.

[21] Lin JT, Wu D, Yang J (2021). Exercising with a six pack in virtual reality: examining the proteus effect of avatar body shape and sex on self-efficacy for core-muscle exercise, self-concept of body shape, and actual physical activity. Front Psychol.

[22] Napolitano MA, Hayes S, Russo G, Muresu D, Giordano A, Foster GD (2013). Using avatars to model weight loss behaviors: participant attitudes and technology development. J Diabetes Sci Technol.

[23] Navarro J, Cebolla A, Llorens R, Borrego A, Baños RM (2020). Manipulating self-avatar body dimensions in virtual worlds to complement an internet-delivered intervention to increase physical activity in overweight women. Int J Environ Res Public Health.

[24] Navarro J, Peña J, Cebolla A, Baños R (2022). Can avatar appearance influence physical activity? User-avatar similarity and proteus effects on cardiac frequency and step counts. Health Commun.

[25] Stein N, Brooks K (2017). A fully automated conversational artificial intelligence for weight loss: longitudinal observational study among overweight and obese adults. JMIR Diabetes.

[26] Stephens TN, Joerin A, Rauws M, Werk LN (2019). Feasibility of pediatric obesity and prediabetes treatment support through Tess, the AI behavioral coaching chatbot. Transl Behav Med.

[27] Tammy Lin JH, Wu DY (2021). Exercising with embodied young avatars: how young vs. older avatars in virtual reality affect perceived exertion and physical activity among male and female elderly individuals. Front Psychol.

[28] Thomas JG, Spitalnick JS, Hadley W, Bond DS, Wing RR (2015). Development of and feedback on a fully automated virtual reality system for online training in weight management skills. J Diabetes Sci Technol.

[29] Thompson DI, Cantu D, Callender C, Liu Y, Rajendran M, Rajendran M, Zhang Y, Deng Z (2018). Photorealistic avatar and teen physical activity: feasibility and preliminary efficacy. Games Health J.

[30] Ahn SJ, Johnsen K, Ball C (2019). Points-based reward systems in gamification impact children's physical activity strategies and psychological needs. Health Educ Behav.

[31] Alves da Cruz MM, Ricci-Vitor AL, Bonini Borges GL, Fernanda da Silva P, Ribeiro F, Marques Vanderlei LC (2020). Acute hemodynamic effects of virtual reality-based therapy in patients of cardiovascular rehabilitation: a cluster randomized crossover trial. Arch Phys Med Rehabil.

[32] Balsa J, Félix I, Cláudio AP, Carmo MB, Silva IC, Guerreiro A, Guedes M, Henriques A, Guerreiro MP (2020). Usability of an intelligent virtual assistant for promoting behavior change and self-care in older people with type 2 diabetes. J Med Syst.

[33] Behm-Morawitz E, Lewallen J, Choi G (2016). A second chance at health: how a 3D virtual world can improve health self-efficacy for weight loss management among adults. Cyberpsychol Behav Soc Netw.

[34] Bickmore TW, Schulman D, Sidner C (2013). Automated interventions for multiple health behaviors using conversational agents. Patient Educ Couns.

[35] Chin J, Quinn K, Muramatsu N, Marquez D (2020). A user study on the feasibility and acceptance of delivering physical activity programs to older adults through conversational agents. Proc Hum Factors Ergon Soc Annu Meet.

[36] Dhinagaran DA, Sathish T, Soong A, Theng Y, Best J, Tudor Car L (2021). Conversational agent for healthy lifestyle behavior change: web-based feasibility study. JMIR Form Res.

[37] Dol A, Bode C, Velthuijsen H, van Strien T, van Gemert-Pijnen L (2021). Application of three different coaching strategies through a virtual coach for people with emotional eating: a vignette study. J Eat Disord.

[38] Duncan-Carnesciali J, Wallace BC, Odlum M (2018). An evaluation of a diabetes self-management education (DSME) intervention delivered using avatar-based technology: certified diabetes educators' ratings and perceptions. Diabetes Educ.

[39] Hahn L, Rathbun SL, Schmidt MD, Johnsen K, Annesi JJ, Ahn SJ (2020). Using virtual agents and activity monitors to autonomously track and assess self-determined physical activity among young children: a 6-week feasibility field study. Cyberpsychol Behav Soc Netw.

[40] Hickman RL, Clochesy JM, Alaamri M (2021). Effects of an eHealth intervention on patient-provider interaction and functional health literacy in adults with hypertension. SAGE Open Nurs.

[41] Johnson-Glenberg MC, Hekler EB (2013). "Alien health game": an embodied exergame to instruct in nutrition and MyPlate. Games Health J.

[42] Klaassen R, Bul KC, Op den Akker R, van der Burg GJ, Kato PM, Di Bitonto P (2018). Design and evaluation of a pervasive coaching and gamification platform for young diabetes patients. Sensors (Basel).

[43] Kowalska M, Gładyś A, Kalańska-Łukasik B, Gruz-Kwapisz M, Wojakowski W, Jadczyk T (2020). Readiness for voice technology in patients with cardiovascular diseases: cross-sectional study. J Med Internet Res.

[44] Krishnakumar A, Verma R, Chawla R, Sosale A, Saboo B, Joshi S, Shaikh M, Shah A, Kolwankar S, Mattoo V (2021). Evaluating glycemic control in patients of south Asian origin with type 2 diabetes using a digital therapeutic platform: analysis of real-world data. J Med Internet Res.

[45] Kuo H, Lee C, Chiou W (2016). The power of the virtual ideal self in weight control: weight-reduced avatars can enhance the tendency to delay gratification and regulate dietary practices. Cyberpsychol Behav Soc Netw.

[46] Li BJ, Lwin MO, Jung Y (2014). Wii, myself, and size: the influence of proteus effect and stereotype threat on overweight children's exercise motivation and behavior in exergames. Games Health J.

[47] Mestre DR, Ewald M, Maiano C (2011). Virtual reality and exercise: behavioral and psychological effects of visual feedback. Stud Health Technol Inform.

[48] Mitchell S, Bragg A, Gardiner P, De La Cruz B, Laird L (2022). Patient engagement and presence in a virtual world world diabetes self-management education intervention for minority women. Patient Educ Couns.

[49] Napolitano MA, Harrington CB, Patchen L, Ellis LP, Ma T, Chang K, Gaminian A, Bailey CP, Evans WD (2021). Feasibility of a digital intervention to promote healthy weight management among postpartum African American/Black women. Int J Environ Res Public Health.

[50] Naylor JB, Patton BJ, Barkley JE (2020). VO2, liking, and relative reinforcing value of cooperative and competitive exergame play in young children. Int J Exerc Sci.

[51] Piao M, Ryu H, Lee H, Kim J (2020). Use of the healthy lifestyle coaching chatbot app to promote stair-climbing habits among office workers: exploratory randomized controlled trial. JMIR Mhealth Uhealth.

[52] Ruiz JG, Andrade AD, Anam R, Aguiar R, Sun H, Roos BA (2012). Using anthropomorphic avatars resembling sedentary older individuals as models to enhance self-efficacy and adherence to physical activity: psychophysiological correlates. Stud Health Technol Inform.

[53] Ruiz JG, Andrade AD, Anam R, Lisigurski M, Karanam C, Sharit J (2014). Computer-based programmed instruction did not improve the knowledge retention of medication instructions of individuals with type 2 diabetes mellitus. Diabetes Educ.

[54] Ruiz JG, Andrade AD, Karanam C, Krishnamurthy D, Niño L, Anam R, Sharit J (2016). The communication of global cardiovascular risk by avatars. Stud Health Technol Inform.

[55] Santini S, Stara V, Galassi F, Merizzi A, Schneider C, Schwammer S, Stolte E, Kropf J (2021). User requirements analysis of an embodied conversational agent for coaching older adults to choose active and healthy ageing behaviors during the transition to retirement: a cross-national user centered design study. Int J Environ Res Public Health.

[56] Sinoo C, van der Pal S, Blanson Henkemans OA, Keizer A, Bierman BP, Looije R, Neerincx MA (2018). Friendship with a robot: children's perception of similarity between a robot's physical and virtual embodiment that supports diabetes self-management. Patient Educ Couns.

[57] Srivastava P, Verma A, Geronimo C, Button TM (2019). Behavior stages of a physician- and coach-supported cloud-based diabetes prevention program for people with prediabetes. SAGE Open Med.

[58] Strombotne KL, Lum J, Ndugga NJ, Utech AE, Pizer SD, Frakt AB, Conlin PR (2021). Effectiveness of a ketogenic diet and virtual coaching intervention for patients with diabetes: a difference-in-differences analysis. Diabetes Obes Metab.

[59] Thompson D, Callender C, Gonynor C, Cullen KW, Redondo MJ, Butler A, Anderson BJ (2019). Using relational agents to promote family communication around type 1 diabetes self-management in the diabetes family teamwork online intervention: longitudinal pilot study. J Med Internet Res.

[60] Thompson D, Cullen KW, Redondo MJ, Anderson B (2016). Use of relational agents to improve family communication in type 1 diabetes: methods. JMIR Res Protoc.

[61] To QG, Green C, Vandelanotte C (2021). Feasibility, usability, and effectiveness of a machine learning-based physical activity chatbot: quasi-experimental study. JMIR Mhealth Uhealth.

[62] Tongpeth J, Du HY, Clark RA (2018). Development and feasibility testing of an avatar-based education application for patients with acute coronary syndrome. J Clin Nurs.

[63] Friederichs S, Bolman C, Oenema A, Guyaux J, Lechner L (2014). Motivational interviewing in a web-based physical activity intervention with an avatar: randomized controlled trial. J Med Internet Res.

[64] Hahn L, Schmidt MD, Rathbun SL, Johnsen K, Annesi JJ, Ahn SJ (2020). Using virtual agents to increase physical activity in young children with the virtual fitness buddy ecosystem: study protocol for a cluster randomized trial. Contemp Clin Trials.

[65] Brown SJ, Lieberman DA, Germeny BA, Fan YC, Wilson DM, Pasta DJ (1997). Educational video game for juvenile diabetes: results of a controlled trial. Med Inform (Lond).

[66] Thompson D, Cantu D, Rajendran M, Rajendran M, Bhargava T, Zhang Y, Chen C, Liu Y, Deng Z (2016). Development of a teen-focused exergame. Games Health J.

[67] Moher D, Liberati A, Tetzlaff J, Altman DG, PRISMA Group (2009). Preferred reporting items for systematic reviews and meta-analyses: the PRISMA statement. PLoS Med.

[68] Aparicio-Ugarriza R, Cuenca-García M, Gonzalez-Gross M, Julián C, Bel-Serrat S, Moreno LA, Breidenassel C, Kersting M, Arouca AB, Michels N, Mouratidou T, Manios Y, Dallongeville J, Gottrand F, Widhalm K, Kafatos A, Molnár D, De Henauw S, Gunter MJ, Huybrechts I (2019). Relative validation of the adapted Mediterranean diet score for adolescents by comparison with nutritional biomarkers and nutrient and food intakes: the healthy lifestyle in Europe by nutrition in adolescence (HELENA) study. Public Health Nutr.

[69] Stavropoulos V, Pontes HM, Gomez R, Schivinski B, Griffiths M (2020). Proteus effect profiles: how do they relate with disordered gaming behaviours?. Psychiatr Q.

[70] Dauchot N (2018). Introduction to the proteus effect. Medium.

[71] Hariton E, Locascio J (2018). Randomised controlled trials - the gold standard for effectiveness research: study design: randomised controlled trials. BJOG.

[72] Bondemark L, Ruf S (2015). Randomized controlled trial: the gold standard or an unobtainable fallacy?. Eur J Orthod.

[73] Yee N, Bailenson J (2007). The proteus effect: the effect of transformed self-representation on behavior. Human Comm Res.

[74] Lyzwinski LN, Caffery LJ, Bambling M, Edirippulige S (2018). Consumer perspectives on mHealth for weight loss: a review of qualitative studies. J Telemed Telecare.

[75] Yanai H, Tomono Y, Ito K, Furutani N, Yoshida H, Tada N (2008). The underlying mechanisms for development of hypertension in the metabolic syndrome. Nutr J.

[76] Franklin SS (2006). Hypertension in the metabolic syndrome. Metab Syndr Relat Disord.

[77] Sparrenberger F, Cichelero FT, Ascoli AM, Fonseca FP, Weiss G, Berwanger O, Fuchs SC, Moreira LB, Fuchs FD (2009). Does psychosocial stress cause hypertension? A systematic review of observational studies. J Hum Hypertens.

[78] Nguyen Q, Dominguez J, Nguyen L, Gullapalli N (2010). Hypertension management: an update. Am Health Drug Benefits.

[79] Swarup S, Goyal A, Grigorova Y, Zeltser R (2021). Metabolic syndrome. StatPearls.

[80] Lee M, Han K, Kim MK, Koh ES, Kim ES, Nam GE, Kwon H (2020). Changes in metabolic syndrome and its components and the risk of type 2 diabetes: a nationwide cohort study. Sci Rep.

[81] Seo EH, Kim H, Kwon O (2019). Association between total sugar intake and metabolic syndrome in middle-aged Korean men and women. Nutrients.

[82] Zając-Gawlak I, Pelclová J, Groffik D, Přidalová M, Nawrat-Szołtysik A, Kroemeke A, Gába A, Sadowska-Krępa E (2021). Does physical activity lower the risk for metabolic syndrome: a longitudinal study of physically active older women. BMC Geriatr.

[83] Bankoski A, Harris TB, McClain JJ, Brychta RJ, Caserotti P, Chen KY, Berrigan D, Troiano RP, Koster A (2011). Sedentary activity associated with metabolic syndrome independent of physical activity. Diabetes Care.

[84] Moyer AE, Rodin J, Grilo CM, Cummings N, Larson LM, Rebuffé-Scrive M (1994). Stress-induced cortisol response and fat distribution in women. Obes Res.

[85] Kandiah J, Yake M, Jones J, Meyer M (2006). Stress influences appetite and comfort food preferences in college women. Nutr Res.

[86] Torres SJ, Nowson CA (2007). Relationship between stress, eating behavior, and obesity. Nutrition.

[87] Wichianson JR, Bughi SA, Unger JB, Spruijt-Metz D, Nguyen-Rodriguez ST (2009). Perceived stress, coping and night-eating in college students. Stress Med.

[88] Yau YH, Potenza MN (2013). Stress and eating behaviors. Minerva Endocrinol.

[89] Chiesa A, Malinowski P (2011). Mindfulness-based approaches: are they all the same?. J Clin Psychol.

[90] Chiesa A, Serretti A (2009). Mindfulness-based stress reduction for stress management in healthy people: a review and meta-analysis. J Altern Complement Med.

[91] Labee EE (2011). Psychology Moment by Moment: A Guide to Enhancing Your Clinical Practice with Mindfulness and Meditation.

[92] O'Reilly GA, Cook L, Spruijt-Metz D, Black DS (2014). Mindfulness-based interventions for obesity-related eating behaviours: a literature review. Obes Rev.

[93] Olson KL, Emery CF (2015). Mindfulness and weight loss: a systematic review. Psychosom Med.

